# Attacking Tumors From All Sides: Personalized Multiplex Vaccines to Tackle Intratumor Heterogeneity

**DOI:** 10.3389/fimmu.2019.00824

**Published:** 2019-04-16

**Authors:** Felix L. Fennemann, I. Jolanda M. de Vries, Carl G. Figdor, Martijn Verdoes

**Affiliations:** ^1^Department of Tumor Immunology, Radboud Institute for Molecular Life Sciences, Radboud University Medical Center, Nijmegen, Netherlands; ^2^Department of Medical Oncology, Radboud University Medical Center, Nijmegen, Netherlands; ^3^Division of Immunotherapy, Oncode Institute, Radboud University Medical Center, Nijmegen, Netherlands; ^4^Institute for Chemical Immunology, Nijmegen, Netherlands

**Keywords:** tumor vaccines, personalized vaccines, neoantigens, intratumor heterogeneity, multiplex neoantigen vaccines

## Abstract

Tumor vaccines are an important asset in the field of cancer immunotherapy. Whether prophylactic or therapeutic, these vaccines aim to enhance the T cell-mediated anti-tumor immune response that is orchestrated by dendritic cells. Although promising preclinical and early-stage clinical results have been obtained, large-scale clinical implementation of cancer vaccination is stagnating due to poor clinical response. The challenges of clinical efficacy of tumor vaccines can be mainly attributed to tumor induced immunosuppression and poor immunogenicity of the chosen tumor antigens. Recently, intratumor heterogeneity and the relation with tumor-specific neoantigen clonality were put in the equation.In this perspective we provide an overview of recent studies showing how personalized tumor vaccines containing multiple neoantigens can broaden and enhance the anti-tumor immune response. Furthermore, we summarize advances in the understanding of the intratumor mutational landscape containing different tumor cell subclones and the temporal and spatial diversity of neoantigen presentation and burden, and the relation between these factors with respect to tumor immunogenicity. Together, the presented knowledge calls for the investment in the characterization of neoantigens in the context of intratumor heterogeneity to improve clinical efficacy of personalized tumor vaccines.

## Introduction–a Short History of Tumor Vaccines

The beginnings of immunotherapy date back to the late Nineteenth century. In 1891, the American bone surgeon William B. Coley started to treat cancer patients with bacterial injections with the rationale to stimulate the immune system and thereby enhance tumor cell killing. With varying success “Coley's Toxins” were accepted as treatment for inoperable bone cancers but could never fully be clinically established (1). In 1909, Paul Ehrlich established the concept of protective vaccinations of mice by transplanting them with foreign tumors. He described that in 50–100% of vaccinated mice an acquired tumor-directed immunity could be observed (2). The genetic basis of this type of rejection was discovered in 1914 by Clarence Little (3), and later linked to histocompatibility antigens on transplanted tumors by Gorer et al. (4). In line with these findings, Foley (5) and Prehn et al. (6) investigated the mechanism of protection in mice against carcinogen-induced sarcomas after rechallenge with the same tumor, thereby formulating the rational of using tumor tissue as a vaccine. In the following years more pieces of the immunological puzzle were solved finally culminating in the concept of immunological surveillance, formulated by Burnet in 1970, further justifying the use of cancer vaccines (7).

Nowadays, it is well-established that tumor vaccines can effectively mount an anti-tumor immune response. These vaccines can be comprised of whole tumor lysates, recombinant tumor proteins, tumor antigen derived epitope peptides, or antigen encoding mRNA and DNA (8). Once injected, dendritic cells (DCs) play a crucial role by taking up the vaccine and presenting the vaccine-derived tumor epitope in the context of major histocompatibility class (MHC) I or II complexes to CD8^+^ or CD4^+^ T cells, respectively (9, 10). In turn, ideally tumor specific CD8^+^ T cells will be activated, proliferate and infiltrate into the tumor to exert cytotoxic functions. CD4^+^ T cells are skewed toward T helper cell subsets and support the anti-tumor immune response by the release of cytokines (11, 12) or tumor cell killing (13).

Despite promising results obtained in 1995 with a DC vaccine pulsed *ex vivo* with the melanoma tumor antigen 1 (MAGE-1) (14, 15), it was not until 2010 before the first DC-based vaccine Sipuleucel-T was approved by the Food and Drug Administration (FDA). This revitalized the tumor vaccine field resulting in the initiation of clinical trials to test new formulations and delivery methods of tumor vaccines across multiple types of cancers.

Especially, recent developments in the understanding of the nature of tumor antigens have attributed to the improvement of tumor vaccines. Together with new insights into the mutational landscape of tumors and their evolution these findings are instrumental for the rise of novel, multiplex and personalized tumor vaccines.

## The Importance of Antigen-Specificity for Tumor Vaccines

Since Edward Tyzzer coined the term “somatic mutation” in 1916 for describing “modifications of the somatic tissue” that determine foreignness and antigenicity of a transplanted tumor, it took until 1991 to discover the first tumor antigen MAGE-1(16, 17). MAGE-1 was shown to be expressed on patient-derived melanoma cells and in immune privileged sites, such as testis, hence the name cancer/testis antigen (CTA), and therefore qualified as a good target for immunotherapy (17). Although CTAs are specific targets on tumor cells and therefore classify as candidates for tumor vaccines, their expression is limited to a small number of tumors and patients (18). In contrast to cancer/testis antigens, tumor-associated antigens (TAAs), such as gp100, tyrosinase or EGFR, are overexpressed on tumor cells and are shared in a bigger patient population (19). Their concurrent expression on healthy cells however, will result in a weaker antigen-specific immune response, due to negative selection as a consequence of central tolerance. Furthermore, DCs and regulatory T cells will dampen the immune response by inducing peripheral tolerance and inhibiting effector T cells (20). Together with the potential of inducing auto-immune reactions, these features underline that TAAs are not ideal candidates for effective tumor vaccination and that therapy targets are preferably expressed exclusively on tumor cells.

In 1994, the first report of such tumor-specific antigen (TSA) was published, being a mutated version of the membrane protein Connexin-37 in Lewis lung carcinoma (21). In the following years the rise of next-generation sequencing techniques led to the discovery of more TSAs or so called neoantigens. Neoantigens are seen as highly specific tumor antigens that arise due to somatic mutations exclusively in tumor cells while being absent in healthy cells [for extensive reviews about neoantigens see (22, 23)]. Except of mutations in driver genes, such as isocitrate dehydrogenase 1 (IDH1)(24) or KRAS(25) and in a rare form of hereditary colon cancer, called Lynch syndrome(26), neoantigens are not shared between individual patients and can have differential expression in tumor clones within one patient, as will be discussed later. Moreover, the load of neoantigens has been positively correlated with the presence of tumor-infiltrating, neoantigen-specific T cells and a good prognosis for checkpoint inhibitor therapy and survival across different types of cancers (27, 28). In turn researchers have made use of sequencing and peptide-based assays combined with computational filtering and prediction algorithms for the selection of candidate neoantigens to design the first generation of personalized tumor vaccines (29, 30). An example of such, is a point mutation in the gene encoding IDH1, which is shared by about 70% of diffuse grade II and III glioma patients, as mentioned above. Using the mutant IDH1 as synthetic long peptide vaccine, Schumacher et al. observed reduced tumor growth in vaccinated mice carrying tumors with the IDH1 point mutation compared to mice carrying IDH1 WT tumors (Table 1) (24).

**Table 1:** Summary of neoantigen vaccine studies ordered by the amount of neoepitopes that are incorporated in the vaccine.

**Tumor type**	**Organism**	**Neoantigen identification (origin, method)**	**Neoantigen prediction**	**Vaccine format**	**Amount of neoantigens used for vaccination**	**Year**	**Publication**
Melanoma, B16F10	Mouse	(29) Mass spectronomy	-	PLGA capturing endogenous neoantigen containing proteins	n.m.	2017	(31)
Colon cancer CT26, TC-1, melanoma B16F10	Mouse	(29)	-	SLP in polyethyleneimine mesoporous silica microrods	n.m.	2018	(32)
Colon cancer MC-38, TC-1	Mouse	(33)	-	Ferritin nanoparticle neoantigen conjugates	1 (TC-1) 3 (MC-38)	2019	(34)
Sarcoma, A2.DR1	Mouse	(Most frequent point mutation in glioma)	-	SLP	1	2014	(24)
Colon cancer CT26, TC-1, melanoma	Mouse	(29)	-	RNA lipoplex	1	2016	(35)
Colon cancer, MC-38	Mouse	(33)	-	RNA-DNA nanostructures	1	2018	(36)
Sarcoma, d42m1-T3 and F244	Mouse	Complement DNA capture sequencing,	3x MHCI epitope binding, processing by immunoproteasome	SLP	2	2014	(37)
Melanoma, B16F10	Mouse	B16F10 cells, DNA/RNA sequencing	Expression, Location, mutation type, immunogenicity	SLP	2	2012	(29)
Melanoma B16F10, colon cancer MC-38	Mouse	(29, 33)	-	RNA nanodisc	2	2016	(38)
Colon cancer MC38	Mouse	Whole exon and RNA sequencing, Mass spectronomy	netMHC binding prediction, solvent exposure in MHC	SLP	3	2014	(33)
Melanoma, colon cancer, HPV E6/E7	Mouse	(29)	-	PC7A nanoparticle	3	2017	(39)
Melanoma B16F10	Mouse	Exome/RNA sequencing	MHCII Class binding	mRNA	5	2015	(30)
Melanoma	Human	Resected tumor, Exome sequencing	Binding to HLA-A, cDNA expression	SLP	7	2015	(40)
Stage III/IV Melanoma	Human	Tumor biopsy, Exome and RNA sequencing	Binding affinity to HLA class II and expression of mutation encoding RNA, and HLA class I binding	mRNA	10	2017	(41)
Lewis lung carcinoma, TC-1, ovarian cancer ID8	Mouse	Cell lines and lysed tumor, Whole exome and RNA sequencing	Binding affinity to HLA class I and II, proteasomal processing	Plasmid DNA	12	2019	(42)
Melanoma Stage IIIB/C Stage IVM1a/b	Human	Whole exon sequencing and RNA sequencing	Binding to HLA-A and -B	SLP	20	2017	(43)
Glioblastoma	Human Phase I/Ib	Whole exon sequencing and RNA sequencing	Binding to HLA-A and -B	SLP	20	2019	(44)

*n.m, not mentioned*.

## From Single to Multiplex Personalized Neoantigen Vaccines

The IDH1 synthetic long peptide is an example of a rationally designed neoantigen vaccine based on a tumor-specific point mutation shared by a large patient population with a mildly immunogenic tumor. For more immunogenic tumors with higher mutational load such as melanoma, high throughput genome screens are needed. One of the first studies applying this strategy used massive parallel sequencing of mouse tumor and healthy tissue combined with RNA expression profiling and immunogenicity tests to obtain potential neoantigen sequences for vaccination purposes. Eventually, vaccinations were performed with two to five neoantigens in the form of synthetic long peptides or mRNA. These led to significant delay of tumor growth and protection of mice in a prophylactic (29) or therapeutic setting (29, 30). Predicted mutations for the B16F10 melanoma, CT-26 colon cancer or 4T1 mammary carcinoma models by Castle and Kreiter et al. were subsequently used and extended to generate neoantigen vaccines targeting more than one epitope simultaneously, and delivered as synthetic long peptide (43), mRNA (41), by carrier molecules such as nanoparticles (31, 34, 36, 39), nanodiscs (38, 45) or other modalities (33, 35, 46) (Table 1). In addition to this, in pre-clinical mouse studies, the concurrent use of checkpoint inhibitors programmed cell death-1 (PD-1) or cytotoxic T-lymphocyte-associated protein-4 (CTLA-4) at the time of vaccination worked synergistically and enhanced the treatment outcome (31, 34, 39, 45).

In clinical studies of late stage melanoma patients, multiplex personalized neoantigen vaccines have achieved significant results in clinical studies of late stage melanoma patients, as nicely summarized by Hellmann and Snyder (47). Recently, a similar approach has been presented for phase Ib glioblastoma patients. These patients received personalized neoantigen vaccines, covering 20 neoepitopes in the form of long peptides, based on mutational profiling and RNA expression analysis of surgically resected tumors. Although all patients died due to progressive disease, neoantigen-specific CD8^+^ and CD4^+^ T cells could be observed which were able to infiltrate into the tumor (44). Finally, also DNA has been used as a delivery vector for encoding neoantigen vaccines. Duperret et al. used a combination of intramuscular injection and electroporation of plasmids with strings of up to twelve 9-mer neoepitopes, derived from lung carcinoma or ovarian cancer. Neoantigen-specific immune response were predominantly guided by CD8^+^ T cells and resulted in a delay of tumor growth and increased survival in prophylactically or therapeutically vaccinated mice (42).

Concurrently, a general trend that can be observed in recent studies is an increase in neoepitope incorporation into tumor vaccines (Table 1). This is mainly the result of the increasing improvements of next-generation sequencing and computational tools for prediction of neoantigens, providing a more detailed view on the mutational landscape of tumors.

Argumentation supporting multiplex neoantigen vaccines can be found in a more fundamental aspect of tumor evolution which has been elucidated, especially within the last 10 years. It relates to the understanding of the identity of individual tumor cells within specific regions in the tumor mass. Although it is well-known that tumors are heterogeneous, comprised of different cell types, such as immune cells and stromal cells, more knowledge had to be gained at the single cell- and genotypic- level of malignant cells. As such, it was elucidated that a certain degree of tumor cell evolution takes place within one tumor leading to the formation of subclones separated not only spatially, but also by mutational patterns (48–52). The latest key findings on the deciphering of intratumor heterogeneity (ITH), its relation to neoantigen expression and its effect on the immune system and immunotherapy present an essential milestone toward the next generation of multiplex personalized neoantigen vaccines and offer an outlook on the challenges we face in the future.

## Intratumor Heterogeneity Challenging Multiplex Personalized Neoantigen Vaccines

The concept of ITH was first introduced in the 1970's by Prehn et al. who investigated the immunogenicity of methylcholanthrene-induced murine sarcomas. Paired cancer cell subclones from different regions of a primary tumor showed differential induction of immune responses after transplantation into recipient mice. These differences were thought to be caused by distinct antigenicity and immunogenicity of subclones, hence a heterogeneous distribution within one tumor (53). In the following years other groups extended this knowledge by delineating the types of immune responses toward heterogeneous tumors based on subclonal expression of tumor antigens (54).

The first human study to investigate the extent of ITH within one tumor mass was performed in 2012 by Gerlinger et al. (48). In this study, several biopsies from a patient-derived primary renal-cell carcinoma were analyzed by whole-exome sequencing and aligned to healthy tissue. Next to several shared mutations between different subclones, ca. 23% of the mutations were only found in specific regions of the tumor. Strikingly, a single biopsy of that same tumor only covered around 55% of the total mutational diversity, underlining the need for multi-region sampling. Tracing the order of mutations in different subclones revealed that they develop in a branching fashion from the primary tumor clone, harboring the driver mutation, rather than in a linear model. Remarkably, these differentially branched subclones harbored different mutations in the same gene which suggests a mode of convergent evolution (48). These findings emphasize the importance of multi-region sampling of tumor samples, as it can explain the mutational ancestry of a tumor and thereby aid in the selection of neoantigens for tumor vaccination, which ideally target mutations from the trunk of the phylogenetic tree.

Besides being able to reconstruct the mutational history, it is also important to correlate this to the developmental stage of the tumor as shown by De Bruin et al. (49). In patients with non-small cell lung cancer (NSCLC) mutational events in the primary tumor coupled to known driver genes could be identified in the context of tobacco-induced carcinogenesis, bearing typical C>T transitions in early development. Mutations in driver genes were also observed in subclones of later development, however these clones also acquired other somatic mutations indicative of a branched evolution and supporting the idea of ITH in NSCLC. Knowing that these tumors carry driver mutations in late stage development, in different regions of the tumor, emphasizes the benefit of multi-dimensional sampling and sequencing for developing tumor vaccines that target these driver mutations (49). Importantly, in this study <5% of the tumor tissue could be analyzed, which probably underestimates the extend of observed ITH.

**Figure 1:**
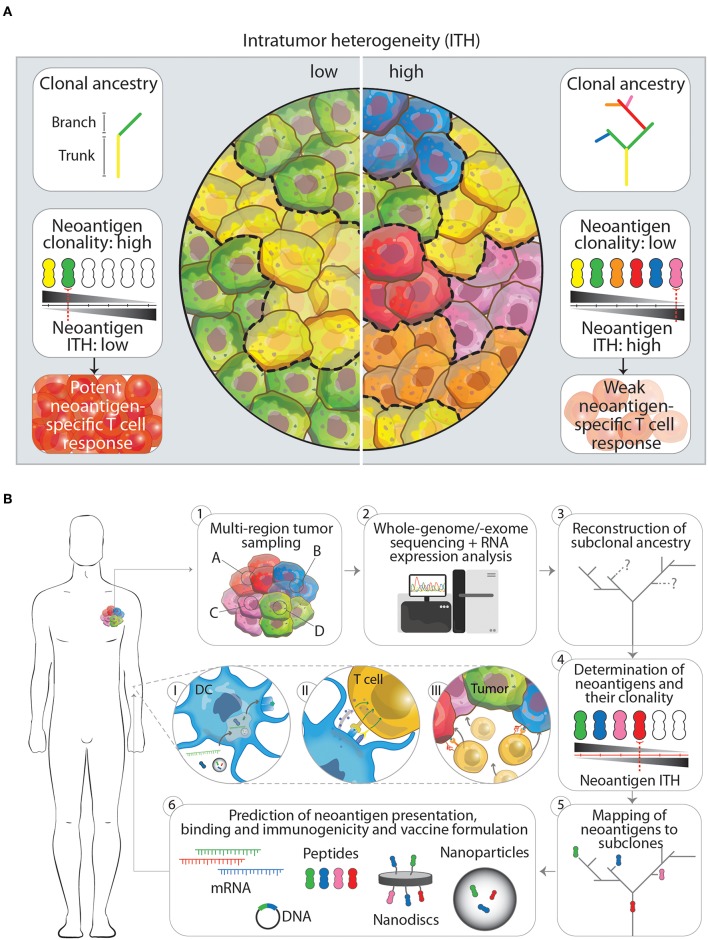
**(A)** The impact of low and high intratumor heterogeneity (ITH) on clonal ancestry, neoantigen clonality and T cell responses. Tumors that show low ITH (left panel) typically have few branching mutations as indicated in the clonal ancestry panel. In turn, more cells in the tumor harbor the same mutation, which is potentially translated and presented on the cell as a neoantigen. The overall neoantigen clonality (the number of cells that express one specific neoantigen, indicated by black-gray triangle) is therefore higher, leading to a lower neoantigen ITH and subsequently in a better neoantigen-specific T cell response. Tumors that have a high ITH in contrast (right panel), show more branching mutations leading to an increased amount of neoantigens expressed. Having more subclones with specific neoantigens however decreased the neoantigen clonality and increases neoantigen ITH. This will result in a weaker neoantigen-specific T cell response. **(B)** Workflow for the designing of next generation multiplex neoantigen vaccines addressing ITH (1–6). (1) Ideally, the generation of multiplex neoantigen vaccines starts with multi-region tumor sampling by preferentially, non-invasive techniques. (2) Acquired data will then be analyzed by whole-genome/-exome sequencing for detection of mutations and RNA expression analysis to infer whether these mutations are located within transcribed regions. (3, 4) From this the subclonal ancestry can be inferred to determine the overall neoantigen clonality and ITH. (5) By mapping found neoantigens to subclones in the tumor and the ancestral tree, target neoantigen can be chosen that are located in the trunk and/or branching regions. (6) Finally, state-off the art prediction algorithms can supplement the aforementioned workflow to cross-validate found neoantigen vaccine candidates that will be incorporated in the final vaccine or vaccine carrier. Panels I-III depict the *in vivo* processing of multiplex neoantigen vaccines leading to a multi-angled anti-tumor T cell response. (I) After injection of multiplex neoantigen vaccines dendritic cells (DCs) will take up and process the vaccine and present antigenic epitopes on the cell surface complexed with MHC molecules. (II) Subsequently, T cells will interact with DCs via T cell receptor-MHC interaction and co-stimulatory molecules and will be further activated under the influence of cytokines. (III) Effector T cells will finally perform cytotoxic effector functions targeting several subclones in the heterogenous tumor.

As growing evidence suggests that diverse sets of mutations occur in subclones in distinct regions of one tumor, McGranahan et al. asked to what extent these mutations translate into neoantigens and how neoantigen ITH (NITH) relates to the anti-tumor immune response (50). Analysis of neoantigen burden and NITH in single biopsies from roughly 200 cases of different types of lung cancers was performed. Using whole-genome and -exome sequencing and bioinformatic processing revealed that a high clonal neoantigen burden (upper quartile of total neoantigen burden) combined with a low NITH (smaller than 1%) correlates with a longer survival in lung adenocarcinoma patients. In contrast, a lower neoantigen clonality (higher NITH) characterized the tumor as more heterogeneous which correlates with a shorter survival (Figure 1A). More homogeneous tumors showed genetic signatures of an inflamed or hot tumor microenvironment with upregulated genes for antigen presentation, T cell migration and effector functions next to inhibitory molecules such as programmed death receptor ligand 1 (PD-L1). As a consequence to this environment, active interferon γ, granzyme B, H and A producing, PD-L1 and lymphocyte activation gene 3 (LAG-3) expressing CD8^+^ T cells specific to clonal neoantigens could be identified within the tumors by MHC multimer staining and flowcytometric analysis. Whether earlier in the development of the tumor it was more heterogeneous, homogenized by initial neoantigen specific T cell infiltration, leading to attraction of more immune cells and an inflamed tumor microenvironment, is however difficult to investigate due to the lack of samples from these earlier developmental stages of the tumor. The observation that these tumors harbored an inflamed, PD-1/L1 expressing microenvironment was the rationale to inhibit PD-1 by checkpoint immunotherapy, which resulted in a clinical benefit for patients with these inflamed tumors. In the same study, similar results have been obtained in a melanoma patient cohort treated with PD-1 checkpoint immunotherapy, where patients with high clonal neoantigen burden and low NITH showed prolonged survival (50). Another example supporting combination of multiplex neoantigen vaccines and checkpoint immunotherapy is provided by two clinical studies of Ott et al. (43) and Sahin et al. (41). In these studies, stage III-IV melanoma patients are initially treated with RNA -or long peptide-based multiplex neoantigen vaccines (Table 1). While most of the patients experienced progression free survival as consequence of neoantigen specific T cell infiltration into the tumor, some showed recurrent disease during multiplex neoantigen vaccination. In these cases, combinatorial treatment with PD-1 blocking antibodies was able to remove tumor mediated immunosuppression and unleash neoantigen-specific T cells that were generated by the vaccine (41, 43).

The discovery that checkpoint immunotherapy results in prolonged survival once neoantigen-specific cytotoxic T cells have infiltrated these tumors, presents a rationale to combine multiplex neoantigen vaccination with checkpoint immunotherapy for tumors with low NITH.

Based on the aforementioned clinical examples a sequential treatment with first multiplex neoantigen vaccines and then, if needed, checkpoint therapy can be suggested to reduce the amount of patients that are unnecessarily treated with checkpoint inhibitors.

As already briefly touched upon above, to what extent neoantigen-specific immune responses shape the heterogeneity of a tumor throughout tumorigenesis by targeting dominant subclones and whether this can lead to tumor escape of untargeted clones remains to be determined.

By applying multi-color barcoding of male Eμ-myc lymphoma cells, Milo et al. studied tumorigenesis and subclonal distribution in a metastatic mouse model (51). When injected in male recipient mice, the differentially colored tumor cells seeded in different proximal niches, ultimately resulting in equally heterogeneous tumors, demonstrating equal survival and outgrowth of the injected barcoded tumor cells. When these cells were injected in female recipient mice, homogeneous tumors with one or two dominant colors established in a CD8^+^ T cell dependent manner. Part of the explanation for this observation can be found in the expression of Y-chromosome derived H-Y antigens, which induced an antigen-specific T cell-mediated immune response. However, the injected mix of color coded tumor cells contained up to 25% non-immunogenic cells due to a loss of the Y-chromosome, suggesting additional clonal reduction as a result of epitope spreading, ultimately resulting in homogeneous Y chromosome deficient tumors. Additionally, whole genome exome-sequencing was applied in this system to infer whether the neoantigen repertoire is narrowed as a consequence of the anti-tumor immune response. In line with the reduction in color-coded subclones in female recipients also NITH was reduced, underlining that the immune system actively shapes subclone diversity and NITH during immunosurveillance, resulting in the evolution of one or few escaping subclones (51).

In a first attempt to study the contribution of neoantigen immunogenicity in the emergence of dominant tumor cell subclones Gejman et al. developed an artificial antigen-presentation system allowing the construction of heterogeneous tumors, expressing up to five thousand defined artificial MHCI neoepitopes (52). Looking at the clearance of immunogenic subclones within a largely heterogeneous tumor in mice, it was revealed that the immune system was incapable of elimintating small clonal fractions of immunogenic subclones. It appeared that the percentage of neoepitope subclonal tumor cell representation is an important determinant for its clearance. This critical subclonal percentage seemed to differ between individual neoepitopes (52). The exact mechanisms behind the persistence of tumor cell subclones, although partly assigned to absence of antigenicity or clonality, remains to be elucidated in more detail. These two studies emphasize the need for controlled systems to investigate the dynamic process of immunosurveillance in the context of heterogeneous tumors with known mutations or neoepitopes to determine how the immune system can be used to reduce subclone diversity and ultimately enable the total clearance of the tumor.

## Conclusion

The discovery of neoantigens and their use as tumor vaccines generated a lot of momentum in the tumor vaccination field. Personalized neoantigen vaccines hold promise in generating specific anti-tumor immune responses and durable survival benefits as emphasized by several pre-clinical and clinical studies. Especially in the last 2 years, vaccines comprising of not one, but several neoepitopes, so called multiplex neoantigen vaccines, have been developed in order to successfully increase the breadth of the anti-tumor immune response.

A rationale that supports this development is obtained from recent insights into the dynamic evolution of tumors. This evolution is characterized by the time-dependent acquisition of region-specific mutations and leads to the emergence of genetically distinct tumor cell subclones within one tumor, as shown by multi-region sampling and massive parallel sequencing. These subclone-specific mutations define the neoantigen clonality, burden and therefore the total NITH, which in turn affects the potency of immunosurveillance. Tumors with a high neoantigen clonality and a low NITH show a better tumor clearance. More heterogeneous tumors with lower neoantigen clonality are more difficult to eradicate (Figure 1A). Depending on this balance between neoantigen clonality and NITH, T cells are able to reduce the diversity of subclones within a tumor and thereby actively shape the ITH. Two important factors which influence the efficacy with which T cells can clear a specific subclone within a heterogeneous tumor seem to be the antigen itself and the percentage of tumor cells expressing this antigen. The exact underlying mechanism has still to be uncovered and can possibly aid us in choosing the right antigens for preventing the emergence of dominant subclones. In the meanwhile, targeting more neoantigens by multiplex neoantigen vaccines is a feasible approach to induce a specific immune response against several subclones in the tumor and thereby address ITH.

## Future Perspectives

Although current studies with multiplex neoantigen vaccines (Table 1) seem to tackle ITH by including more neoepitopes, they are limited by the snapshot of the mutanome acquired by a single biopsy. We believe that the lack of multi-dimensional tumor information in these neoantigen vaccine studies impairs the power of inducing a multi-angled immune response against all the subclones in a tumor. Challenging pre-clinical longitudinal studies of tumorigenesis are needed, taking into account samples from different locations in the tumor at different time points, both before and after treatment. These studies would gain insight in the dynamics of tumor evolution in the context of multiplex neoantigen vaccination. Ultimately, this knowledge could be integrated into the process of designing the next generation of multiplex neoantigen vaccines. Currently, driving neoepitope selection criteria are predicted MHCI binding as well as T cell receptor affinities. We propose a workflow (Figure 1B) starting with the multi-regional NITH acquisition of available biopsies, to obtain multi-dimensional tumor biopsy information, from which dominant clonal neoepitope vaccine candidates could potentially be extrapolated. Attractive, less invasive and practically more feasible alternatives, such as circulating tumor DNA or tumor exosome DNA sequencing, deserve special attention. Varying reports exist about the success of these two techniques in comprehending ITH, which underlines the need for further development of these tools (55–57). Next-generation sequencing and bioinformatic tools that have been developed in the recent years (48, 58–62) will then be an essential asset to acquire genomic sequence information of high, subclonal resolution. With this information the clonal architecture and mutational ancestry of subclones can be reconstructed. Subsequently, neoantigen clonality and burden can be inferred to predict total NITH and map neoantigens to the subclone architecture of the tumor. This dynamic genomic blueprint of the tumor will aid in determining optimal neoantigen candidates for vaccination purposes and can be complemented with bioinformatic prediction algorithms and novel tools to assay T cell reactivity on a large scale (63). Current vaccine production platforms as described earlier, can then facilitate the efficient formulation and delivery of the vaccine to the patient *in vivo*, where DCs will process vaccine content and activate neoantigen specific T cells that will infiltrate and eradicate the tumor (Figure 1B, p. 1–3).

We are convinced that the development of more refined techniques to sample and predict the right neoantigens for vaccination can address ITH, and will be essential to fuel the progress that is currently made with regard to time efficient formulation, design and delivery of multiplex neoantigen vaccines [as extensively reviewed by others (64, 65)]. With these techniques in mind, we could create a more detailed map of the neoantigen clonality in primary tumors and metastasis to determine shared mutations within these regions as shown earlier (48). Along this line, combining multiplex neoantigen vaccines with TAA or CTA epitopes could present a handle to increase the chances of epitope spreading and sensitize the immune system for a priori low abundant neoantigens. A full comprehension of tumor evolution and neoantigen distribution will be the fundament for counteracting the survival of the fittest tumor clone and will pave the way for powerful next-generation multiplex neoantigen vaccines.

## Author Contributions

FF, IdV, CF, and MV contributed to researching the data for the article, discussing the content and to reviewing and editing the manuscript before submission. FF and MV were responsible for writing the article. FF was responsible for figure design.

### Conflict of Interest Statement

The authors declare that the research was conducted in the absence of any commercial or financial relationships that could be construed as a potential conflict of interest.
